# Glycobiology in Human Health and Disease

**DOI:** 10.3390/ijms27072951

**Published:** 2026-03-24

**Authors:** Katrin Mani

**Affiliations:** Department of Experimental Medical Science, Glycobiology Group, Lund University, Biomedical Center A13, SE-221 84 Lund, Sweden; katrin.mani@med.lu.se

## 1. Introduction

Glycobiology has undergone a profound conceptual transformation from a largely descriptive discipline to a central framework for understanding biological regulation in health and disease. Glycans are complex carbohydrate structures covalently attached to proteins and lipids, which form the glycome, a highly dynamic and structurally diverse layer of biological information. Unlike nucleic acids and proteins, glycan biosynthesis is non-template-driven, emerging from the coordinated activity of glycosyltransferases, glycosidases, and modifying enzymes such as deacetylases, sulfotransferases, and epimerases. This enzymatic orchestration generates a vast array of molecular motifs with variations in monosaccharide composition, linkage, branching, and terminal modifications, allowing the glycome to function as a sensor of cellular state and a regulator of molecular interactions [1].

Glycosylation is the most common and diverse post-translational modification in eukaryotes, with complex enzymatic networks that expand proteome diversity and influence nearly every aspect of cellular function [2]. Glycans are distributed at the cell surface, within the extracellular space, and within the cell, where they contribute to receptor organization, signal transduction, intracellular trafficking, and protein folding. At the cell surface, glycans form the glycocalyx, a multifunctional layer that mediates cellular interactions and adhesion while contributing to barrier function.

Recent advances in analytical and computational methods have enabled the description of glycosylation patterns at high resolution, identifying regulatory layers that were previously unknown, enabling the integration of glycan biology into systems-level frameworks [3,4]. The dynamic nature of the glycome emphasizes its responsiveness to developmental, metabolic, and environmental cues, positioning glycans as mediators and coordinators of biological processes. Recent technological breakthroughs and conceptual advances in glycobiology are shaping a new paradigm in which glycan-centric mechanisms are key to understanding human health and disease, from fundamental cellular processes to translational applications [5,6,7]. Technological advances—particularly in mass spectrometry, glycan-specific enrichment strategies, and glycoengineering—have enabled the high-throughput characterization of the glycome, establishing glycomics as a cornerstone of systems biology. When integrated with genomics, transcriptomics, and proteomics, these new approaches have been used to reveal regulatory layers that were previously inaccessible, reshaping our understanding of cellular networks and organismal physiology [4,8].

Although these technological breakthroughs have provided remarkable insights into the glycome, important technical challenges remain. The structural complexity and diversity of glycans, including their isomeric forms and non-template-driven biosynthesis, continue to complicate their comprehensive characterization and quantitative analysis. Limitations in method sensitivity, process standardization, and data interpretation, as well as the need for improved integration with other omics platforms, currently constrain the full realization of glycomics in systems biology and clinical applications. Together, these developments highlight the central role of glycans in modulating cell–cell communication, signaling pathways, and systemic homeostasis, underscoring why glycomics is increasingly indispensable for describing complex biological processes.

Equally important is the translational horizon in this field. Glycan-based biomarkers are highly sensitive indicators of physiological and pathological states, and glycan-targeted interventions—from glycoengineered antibodies to inhibitors of glycan-binding proteins—offer novel therapeutic avenues. The potential of these strategies to refine precision medicine highlights the roles of glycans both mechanistic actors and actionable targets [5].

Integrating glycan biology into mainstream molecular and cellular frameworks promises to not only uncover previously unknown regulatory mechanisms but also inspire innovative approaches to diagnosis and therapy. The field stands at a critical point, with conceptual insights and technological capabilities converging to change our understanding of glycans from supporting actors to central figures in our knowledge of health and disease.

Within this context, the studies in this Special Issue can be appreciated, providing examples of the conceptual and translational advances described above. Each contribution focuses on a different facet of glycobiology, from foundational mechanistic insights to novel translational applications. These papers exemplify how modern glycobiology is reshaping our view of cellular regulation, expanding the boundaries of the field, and providing new opportunities to connect molecular mechanisms with physiological and clinical outcomes. By situating these contributions within the broader landscape of recent technological and conceptual progress, we can appreciate the depth of our current understanding and the directions that lie ahead.

## 2. Insights from This Special Issue

The studies in this Special Issue showcase the growing conceptual and translational depth of glycobiology, highlighting its central role in cellular regulation. Potje et al. (2026) reveal that heparan sulfate chains in the endothelial glycocalyx actively shape vascular reactivity, modulating signaling pathways, with implications for hypertension, atherosclerosis, and vascular inflammation (contribution 1). Mohammed et al. (2025) show that extracellular galectin-3 engages i-linear glycans to drive melanoma aggressiveness, and the I-branching enzyme β1,6 N-acetylglucosaminyltransferase 2 acts as a tumor suppressor. Galectin-3 and i-linear glycans are promising therapeutic targets (contribution 2). Chen et al. (2025) demonstrate that FAM20B, a kinase critical for attaching glycosaminoglycan chains to proteoglycan core proteins, regulates chondrogenic and osteogenic differentiation in the embryonic condyle by controlling Indian hedgehog (IHH) diffusion. Loss of FAM20B accelerates chondrocyte maturation and ossification, highlighting the role of glycans in modulating growth factor signaling (contribution 3). Koike et al. (2024) report that creatine counteracts methylglyoxal-induced carbonyl stress, limiting protein glycation and protecting cells from cytotoxicity, pointing to a metabolic strategy to mitigate glycation-related damage (contribution 4). Demir et al. (2025) highlight lactoferrin, an iron-binding glycoprotein with antimicrobial, immunomodulatory, and antioxidant activities, emphasizing its potential as a functional food additive to enhance food nutrition, safety, and shelf life (contribution 5). Pekdemir et al. (2025) reviewed fucosidosis, a rare lysosomal storage disorder caused by α-L-fucosidase deficiency, illustrating how disruptions in glycan regulation drive systemic pathology, including severe neurodegeneration, and highlighting potential therapeutic avenues (contribution 6). Finally, Zhong et al. (2024) linked protein and lipid sialylation to cell signaling, immune recognition, and disease pathogenesis, emphasizing emerging strategies targeting sialylation in cancer as well as cardiovascular and neurological disorders, showing promise as biomarkers and therapeutic targets (contribution 7).

Together, these studies highlight three overarching themes: the structural and functional diversity of glycans, their dynamic responsiveness to cellular and environmental cues, and their translational potential. Far from peripheral modifications, glycans are as central regulators of physiology and pathology, shaping mechanistic insight and offering avenues for clinical innovation.

## 3. Future Perspectives

The contributions in this Special Issue show that glycobiology has reached a critical point. The field faces challenges and opportunities in deciphering the full and complex glycocode, linking structural motifs to functional outcomes across cellular and organismal contexts.

Integrating glycobiology with systems biology—including single-cell and spatial glycomics—may lead to the uncovering of the heterogeneity of glycosylation, revealing how glycosylation shapes cellular identity, development, and disease. Translationally, glycan-targeted interventions—including glycoengineered proteins, modulators of glycan–lectin interactions, and metabolic strategies—are poised to improve precision medicine, enabling early detection, patient stratification, and tailored therapeutics. Rare disorders such as fucosidosis exemplify how insights from uncommon diseases can inform broader biological principles.

Conceptually, glycans act as sensors and integrators of biological information, responding dynamically to developmental, metabolic, and environmental cues. Understanding this regulatory layer will deepen our knowledge of human health and disease and provide avenues for novel interventions spanning vascular biology, cancer, development, metabolism, nutrition, and immunology.

In summary, glycobiology is no longer a peripheral discipline. The studies presented in this Special Issue exemplify a field in transformation, where mechanistic insight, technological innovation, and translational potential are converging. As we continue to explore the glycome, its dynamic and information-rich nature will redefine how we understand, diagnose, and treat human disease.

Looking ahead, addressing the current technical limitations in glycomics—including structural complexity, quantitative challenges, and integration with multi-omics—will be key to fully realizing the potential of glycobiology in basic research and clinical applications. Continued innovation may expand our ability to translate glycan insights into precision diagnostics and targeted therapies.
